# Understanding Muscle Dysfunction in Chronic Fatigue Syndrome

**DOI:** 10.1155/2016/2497348

**Published:** 2016-02-22

**Authors:** Gina Rutherford, Philip Manning, Julia L. Newton

**Affiliations:** ^1^Institute of Cellular Medicine, Newcastle University, Newcastle NE2 4HH, UK; ^2^Newcastle Hospitals NHS Foundation Trust, UK NIHR Biomedical Research Centre in Ageing and Age Related Disease, Newcastle University, Newcastle NE2 4HH, UK

## Abstract

*Introduction*. Chronic fatigue syndrome/myalgic encephalomyelitis (CFS/ME) is a debilitating disorder of unknown aetiology, characterised by severe disabling fatigue in the absence of alternative diagnosis. Historically, there has been a tendency to draw psychological explanations for the origin of fatigue; however, this model is at odds with findings that fatigue and accompanying symptoms may be explained by central and peripheral pathophysiological mechanisms, including effects of the immune, oxidative, mitochondrial, and neuronal pathways. For example, patient descriptions of their fatigue regularly cite difficulty in maintaining muscle activity due to perceived lack of energy. This narrative review examined the literature for evidence of biochemical dysfunction in CFS/ME at the skeletal muscle level.* Methods.* Literature was examined following searches of PUB MED, MEDLINE, and Google Scholar, using key words such as CFS/ME, immune, autoimmune, mitochondria, muscle, and acidosis.* Results*. Studies show evidence for skeletal muscle biochemical abnormality in CFS/ME patients, particularly in relation to bioenergetic dysfunction.* Discussion.* Bioenergetic muscle dysfunction is evident in CFS/ME, with a tendency towards an overutilisation of the lactate dehydrogenase pathway following low-level exercise, in addition to slowed acid clearance after exercise. Potentially, these abnormalities may lead to the perception of severe fatigue in CFS/ME.

## 1. Introduction

Chronic fatigue syndrome (CFS) also known as Myalgic Encephalomyelitis (ME) is a heterogeneous disorder of unknown aetiology [1]. The condition is characterised by severe disabling fatigue in the absence of alternative diagnosis and is associated with a myriad of other symptoms, including but not limited to postexertional malaise, sleep disturbance, and cognitive dysfunction [1–6]. The prevalence of CFS/ME in the adult population is estimated at around 0.2%–2.6% worldwide [5, 7–9]. For example, the Centre for Disease Control 1994 criteria (CDC4) [10] and the Canadian criteria [11] provide a strict definition and estimate prevalence to be lower (0.2%) than previously reported in a primary care setting whereby a less stringent criteria was applied [5]. In the UK, the condition affects approximately 600,000 individuals, with a peak incidence in the 20–40 age group and a preponderance in females, at a ratio of 6 : 1 [8, 12].

It is important to note that recently there has been intense interest surrounding the naming of the condition. Early in 2015 the Institute of Medicine (IOM) [13] issued a report that proposed a new case definition for CFS/ME, recommending the renaming the illness as Systemic Exertion Intolerance Disease (SEID). This new case definition requires a substantial reduction in ability to complete preillness activities, unrefreshing sleep, postexertional malaise, and either cognitive or orthostatic intolerance. In a recent study conducted by Jason et al. [14], the new SEID criteria were reported to identify a group of patients comparable in size to the Fukuda criteria but a larger group than the Canadian criteria. Additionally, the name was reported to select more patients who had less impairment and fewer symptoms than four-item empiric criteria. Presently, there is considerable debate among scientists as regards which case definition to use for clinical and research purposes. As indicated in the IOM report [13] funding was limited so an inadequate number of studies have focused on the validity and reliability of the case definition [14]. Thus, further research utilising empirical methods is required to fully evaluate the criteria and develop a consensus among researchers, clinicians, and the patient community [14].

One of the primary symptoms of CFS/ME is generalised abnormal muscle fatigue that occurs after relatively mild activity [3, 10, 15]. Fatigue can be defined as a progressive impairment in maximal force generating capacity that develops during muscular activity [16].

Historically, there has been a tendency to draw psychological explanations for the origin of fatigue in CFS/ME; however this model is at odds with patient perceptions of the nature of their condition, with many suggesting a “peripheral” as opposed to “central” origin [15]. For example, patient descriptions of their fatigue regularly cite difficulty in maintaining muscle activity due to perceived lack of energy or through muscle pain, which can be serious enough to lead to the avoidance of exercise [17].

In terms of the pathophysiological basis of peripheral muscle fatigue in CFS/ME, a wide range of potential mechanisms have been suggested. Recent studies [2, 15] using novel magnetic resonance spectroscopy (MRS) techniques have reported distinctive and reproducible muscle bioenergetic abnormality in patients following repeat exercise, contrasting previous work that has reported an absence in increased glycolytic metabolism and lactic acid production in the majority of CFS/ME study participants [18–20]. Additionally, the role of enhanced oxidative stress in CFS/ME has also been reported as a potential mechanism in muscle fatigue, with CFS/ME exhibiting elevated blood oxidative status at rest [21, 22] which is accentuated during exercise [22]. Furthermore, there is evidence to suggest a role of mitochondrial dysfunction in CFS/ME, illustrated by lowered ATP production, impaired oxidative phosphorylation, and mitochondrial damage [23, 24]. Moreover, a key symptom associated with CFS/ME is postexertional malaise (PEM), following physical or emotional stress [25]. Studies have reported exercise in CFS/ME patients to exhibit the potential to amplify preexisting immune dysfunction. Finally, a recent study [26] reported abnormality in AMP-activated protein kinase (AMPK) activation and glucose uptake, when CFS/ME patient skeletal muscle function was assessed via a novel in vitro technique.

Evidence exists to suggest that a wide range of biochemical abnormalities exist at the skeletal muscle level in CFS/ME. This narrative review will explore current evidence regarding the various proposed mechanisms underlying peripheral muscle dysfunction in CFS/ME, which are outlined in Table 1. Ultimately, to gain an improved understanding of muscle dysfunction and the end point of severe disabling fatigue associated with this debilitating condition, improved understanding of the aetiology underlying CFS/ME is pivotal within the field of ageing research, with CFS/ME recognised as an ageing related condition, with symptoms perpetuated amongst older patients.

In terms of methodology, this narrative review examines literature following searches of the electronic databases: PUB MED, MEDLINE, and Google scholar. All papers published up until August 2015 were eligible for inclusion in this review. The search included the following terms: CFS, ME, Immune, autoimmune, mitochondria, muscle, oxidative and nitrosative stress, acidosis, bioenergetics, and AMPK. A total of 435 papers were defined by search, with a total of 67 papers included in this review.

## 2. Central Sensitisation

Central sensitisation is defined as an alteration in the responsiveness of central neurons to input from unimodal and polymodal receptors [44]. Central sensitisation involves a number of top-down and bottom-up mechanisms, which contribute to the hyperresponsiveness of the central nervous system to a variety of stimuli [28]. It is important to note that an alteration in central pathways may impact upon peripheral muscle fatigue.

In terms of central impairment, the perception of fatigue during exercise is not always abnormal and serves an important function during significant physical exertion [2]. For example, in a study conducted by Amann and Dempsey [45] peripheral muscle fatigue was induced in patients and consequently fatiguing muscle was reported to play a pivotal role in the determination of central motor drive and force output. Therefore, suggesting the presence of a feedback signal from peripheral muscle to the central nervous system, to ensure fatigue, is confined to a certain level, preventing damage to the individual. However, it is plausible that peripheral fatigue experienced in CFS/ME is the direct result of excessive signal feedback, leading to a disproportionate perception of fatigue early in the fatiguing process associated with physical activity [15].

Interestingly, evidence exists to suggest the role of central impairment in CFS/ME patients. For example, Whiteside and colleagues [25] reported CFS/ME patients to exhibit a dysfunction of nociceptive inhibition during exercise. This was demonstrated by a decrease in pain threshold following exercise, which is abnormal as pain threshold normally increases during exercise due to a greater release of endogenous opioids and additional inhibitory mechanisms (descending inhibition). This exercise-induced abnormality was also reported in two additional studies [17, 27]. For example, Meeus and colleagues [27] compared CFS/ME patients with chronic pain (*n* = 26), healthy control participants (*n* = 31), and chronic back pain patients (*n* = 21). Participants all completed a submaximal aerobic exercise protocol, which was followed by venous blood sampling (nitric oxide) and algometry (hand, arm, calf, and lower back). Results demonstrated patients with CFS/ME to exhibit lower pain threshold compared to both the healthy control participants and chronic lower back pain participants. Taken together the results demonstrate CFS/ME patients to have a lack of descending inhibition during exercise. The implication of a lack of endogenous inhibition has been suggested to account in part for the PEM experienced by CFS/ME patients.

Evidence also exists to suggest a role of generalised hyperalgesia in CFS/ME as outlined by Nijs and colleagues [28]. It has been suggested that lower pain thresholds in symptomatic locations represents primary hyperalgesia due to sensitised nociceptors within injured peripheral muscle tissue. However, the authors also commented that when pain thresholds are detected in asymptomatic areas, then central sensitisation is at play. Two studies conducted by Vecchiet and colleagues [29] investigated the effect of electrical stimulation of muscle tissue, skin, and subcutis in relation to pain threshold in CFS/ME patients and healthy control participants. Interestingly, in both studies CFS/ME participants reported there to be no significant difference between groups for electrical pain threshold of skin and subcutis. Nevertheless, a much lower electrical pain threshold was observed in all sites of muscle tissue (trapezius, quadriceps, and deltoid) for the CFS/ME group only, illustrative of hyperalgesia in CFS/ME.

## 3. Oxidative and Nitrosative Stress

Enhanced oxidative and nitrosative stress has been reported in CFS/ME patients. For example, studies have demonstrated patients to exhibit excessive production of reactive oxygen species (ROS) following physical exertion, as well as altered resting blood oxidant to antioxidant status [21, 22].

Biochemical markers associated with oxidative stress have also been reported to play a pivotal role in skeletal muscle fatigue [46], which is often cited as a debilitating symptom experienced by CFS/ME patients [22]. Effectively, oxidative and nitrosative stress involve the enhanced production of ROS and reactive nitrogen species (RNS), in addition to other free radicals; these reactive species have the potential to disrupt cell membrane function through lipid peroxidation, as well as damage to functional proteins and DNA. This can ultimately lead to alterations in cell structure and disease initiating mutations [47].

Elevated ROS/RNS exhibit the capacity to profoundly impair mitochondrial function, which has been suggested to be due to the accumulation of oxidative modified mitochondrial proteins, lipids, and DNA [48]. Moreover, these factors exhibit the potential to induce electron transport chain dysfunction, impairment in cellular bioenergetics, and ultimately skeletal muscle fatigue. Furthermore, skeletal muscle is a postmitotic tissue so it is extremely susceptible to mitochondrial oxidative damage; this is due to being terminally differentiated and because of a slow cellular turnover and high metabolic rate [48].

Mitochondria are a major source of ROS and RNS generation in cells and are therefore highly susceptible to oxidative damage. For example, they exhibit reduced levels of antioxidants such as glutathione in comparison to levels found in the cytosol [49]. This relative lack of protection enables mitochondrial DNA (mtDNA) to be damaged, leading to major changes in polypeptide synthesis. Finally, mtDNA repair enzyme activity is considerably lower than found in the nucleus [50]. The combined effects lead to a reduced electron transfer rate with subsequent reduction in the rate of ATP synthesis [51].

In relation to the impact of elevated oxidative stress on muscle dysfunction, muscle specific symptoms of fatigue have been reported to be proportional to the blood levels of a marker of ROS induced lipid peroxidation, thiobarbituric acid reactive substances (TBARS) [21]. TBARS occurs in the serum due to the peroxidation of low-density lipoproteins and oxygen mediated injury of myocyte membranes [29, 52]. Lipid peroxidation of skeletal muscle fibres induces a loss of membrane excitability because of altered activation of K^+^ channels [27, 53]. Additionally, muscle biopsies from healthy adults indicated that the intensity of membrane excitation was proportional to K^+^ efflux measured in plasma [54], with an increase in ROS generation acting to inhibit Na^+^-K^+^ pump activity, thus reducing the K^+^ outflow and muscle membrane excitability [55, 56]. Furthermore, in another study [57] CFS/ME patients were reported to exhibit a reported a loss of Na^+^/K^+^ and Ca^2+^-ATPase pump regulation, as well as alterations in the ryanodine channels within the sarcoplasmic reticulum membrane. This was related to increased fluidity of the sarcoplasmic membrane due to ROS induced formation of lipid hydroperoxides, which the authors suggested to support the hypothesis that sarcolemma conduction system with some aspects of Ca^2+^ transport was negatively influenced in CFS/ME.

Furthermore, in an additional study [22] blood oxidant status and muscle membrane excitability were measured before and after exercise. The case control study compared CFS/ME patients (*n* = 55) and healthy matched controls (*n* = 40). However, within the CFS/ME cohort two subgroups emerged, firstly, those who had reported severe infection (e.g., pneumonia, sepsis, encephalomyelitis, and H1N1 influenza) within 3–7 months preceding onset of CFS/ME symptoms (infected CFS/ME group (I-CFS/ME)); secondly, those who had practiced sport to a high level (>6 hrs per week) for 6 years prior to the onset of CFS/ME symptoms and with no history of severe infection (i.e., noninfected CFS/ME [NI-CFS/ME]). Participants were required to complete a maximal incremental cycle based protocol to reach the point of maximal oxygen uptake. In relation to markers of oxidative stress, blood samples were obtained before, during, and after exercise time points, enabling plasma concentration of TBARS and endogenous antioxidant (reduced ascorbic acid) to be measured. Additionally, action potentials (M-wave) were evoked in the vastus lateralis to explore muscle excitability.

Results demonstrated all CFS/ME patients to exhibit abnormal biochemical and electrophysiological measures indicated by elevated TBARS levels prior to exercise and altered M-wave configuration during and after exercise. These findings are in agreement with other studies [19, 22, 58] which have also reported an elevated blood oxidant status at rest, which was accentuated by exercise, in addition to reduced muscle excitability. For example, one study described enhanced exercise-induced oxidative stress in CFS/ME patients, which was indicated by early changes in TBARS and reduced ascorbic acid in response to incremental exercise. Furthermore, CFS/ME patients also exhibited marked alterations in muscle membrane excitability, indicated by lengthened M-wave duration during the recovery period [58].

However, a pivotal finding of the more recent study [22] was that I-CFS/ME patients exhibited significant accentuation of blood oxidant status at rest, in addition to muscle hypoexcitability at work, as well as significantly reduced K^+^ outflow in response to maximal exercise. Therefore, CFS/ME patients with a history of previous severe infection exhibited greater biological and EMG disorders compared to those who reported completing a high level of physical activity before the onset of fatigue related symptoms. The authors concluded severe infection to act as a stressor responsible for the alteration in blood oxidant status, which may help to explain impaired exercise-induced K^+^ outflow and altered membrane excitability. However, it is important to acknowledge that the NI-CFS patients self-reported their physical activity level for the 6 yrs prior to the onset of CFS/ME symptoms. Therefore, it is possible that CFS/ME patients overestimated their physical activity habits prior to developing CFS/ME symptoms.

Jammes et al. [30] also measured TBARS at rest and following exercise in a CFS/ME patient cohort. Effectively, CFS/ME patients were separated into 4 distinct groups. These were as follows: group I (no relevant previous history), group II (previous high-level sport), group III (previous severe acute infection), and group IV (high-level sport and severe acute infection). Patients were required to complete a maximal cycle protocol before blood samples were collected and plasma was analysed for TBARS and heat shock proteins HSP27 and HSP70. Interestingly, in agreement with other studies [22] the authors reported elevated TBARS at rest in CFS/ME patients compared to controls; however this was only demonstrated in CFS/ME groups II, III, and IV. In contrast to the previously described studies, the authors also measured HSP levels at rest and reported significantly lower levels in all CFS/ME patient groups compared to controls. Following exercise, the peak level of TBARS was increased in CFS/ME groups II, III, and IV. HSP27 and HSP70 were suppressed with the greatest effect in groups III and IV. Therefore, the authors concluded a history of previous stress factors in a CFS/ME patient cohort to be associated with severe and the suppression of protective HSP response to exercise. Similarly, Thambirajah et al. [59] investigated HSP expression at rest and following exercise in a CFS/ME patient cohort. The authors reported basal HSP27 to be elevated in CFS/ME patients compared to controls. Additionally, HSP27 levels decreased immediately after exercise and remained below basal level at day 1 after exercise. In contrast, HSP27 levels were constant at basal and following exercise in the control group. There was also an observed decline in HSP60 and HSP90 in the CFS/ME patient group at 7 days after exercise. The findings were postulated to be evidence of an abnormal or defective adaptive response to oxidative stress in CFS/ME.

Moving on, excessive oxidative and nitrosative stress has also been suggested to be associated with secondary autoimmune responses. Prolonged oxidative and nitrosative stress may cause damage to protein and lipid structures to such an extent that they lose immunogenic tolerance and autoantibodies begin to be formed against them [60]. For example, secondary IgM and IgG mediated autoimmune reactions have been reported to occur in patients with CFS/ME. For example, Maes et al. [31] examined whether CFS/ME was accompanied by a response to neoepitopes and a variety of modified lipids and proteins indicative of damage caused by excessive oxidative and nitrosative stress. Specifically, the authors examined serum antibodies to fatty acids (oleic, palmitic, and myristic acid), by-products of lipid peroxidation (azelaic acid, malondialdehyde, acetylcholine, and S-farnesyl-L-cysteine), and N-oxide modified amino acid in 14 CFS/ME patients and 11 controls. Results indicated mean IgM serum levels directed against oleic, palmitic, and myristic acid, azelaic acid, S-farnesyl-L-cysteine, and the N-oxide derivatives to be significantly greater in CFS/ME patients when compared to controls. In addition, significant and positive correlations were exhibited in the CFS/ME group for serum IgM levels directed against fatty acid malondialdehyde and azelaic acid, N-oxide derivatives, and the severity of illness as measured by the fibro fatigue scale. Furthermore, positive correlations were also reported for the aforementioned products and measures of muscle tension and fatigue. Therefore, it would appear that autoimmune responses as a consequence of oxidative damage to endogenous lipid epitopes could play a pivotal role in CFS/ME associated muscle fatigue.

## 4. Mitochondrial Dysfunction

There is evidence to suggest that mitochondrial dysfunction plays a key role in CFS/ME aetiology. Lowered ATP production, impaired oxidative phosphorylation, and mitochondrial damage have been reported in patients with CFS/ME [23, 24]. Moreover, CFS/ME patients share common skeletal muscle symptoms associated with diseases linked to mitochondrial dysfunction, for example, muscle pain, fatigue, and cramping [61, 62].

There is accumulating evidence to suggest that abnormally high lactate levels and intracellular acidosis exhibited in patients with CFS/ME are the result of impaired mitochondrial function [61, 63]. For example, CFS/ME patients exhibit profound and sustained intracellular acidosis of the peripheral musculature following relatively low-level exercise. This results in a decreased anaerobic threshold (AT) due to an overutilisation of the lactate dehydrogenase pathway [15, 41]. Upon the point of exhaustion CFS/ME patients have been reported to exhibit intracellular ATP concentrations that are lower than those found in nondiseased control participants, which could be indicative of dysfunction in oxidative metabolism. Moreover, in a recent review [61] the authors concluded the response to exercise exhibited by CFS/ME patients to be typical of that reported in individuals with mitochondrial disease. Additionally, there were also a number of similarities between symptoms of mitochondrial disease and the physiosomatic symptoms of CFS/ME. For example, muscle pain, cramps, weakness, and myalgia's [54, 62, 64].

Mitochondrial dysfunction in CFS/ME may be explained by not only elevated oxidative and nitrosative stress but also increased immune-inflammatory stress pathways [65]. Interestingly, chronic low-grade inflammation in CFS/ME has been demonstrated through increased levels of proinflammatory cytokines (IL-1, TNF-*α*) and a movement towards a Th2 dependant immune response, in addition to inflammatory mediators including NF-*κ*B and elastase [64]. Further evidence of elevated oxidative and nitrosative stress has also been demonstrated by increased isoprostane levels, peroxides, and protein carbonyl levels, indicating damage to lipids, protein, and mT as previously discussed [22].

In relation to immune dysfunction, one study examined cytokine networks in 40 female CFS/ME patients and 40 case matched controls [32]. The authors examined a total of 16 cytokines and results revealed a diminution of Th1 and Th17 function and a movement towards Th2 type immunity. Similarly, several other groups have reported a shift for a Th1 to Th2 cytokine profile in CFS/ME patients [33, 34, 66]. For example, Skowera and colleagues [34] examined the frequency of type 1 and type 2 regulator CD4 and CD8 T cells in 35 patients with CFS. Results illustrated a bias towards a Th2 immune response.

Elevated levels of the inflammatory mediator NF-*κ*B [67, 68] have also been reported in CFS/ME. NF-*κ*B is a major upstream intracellular mechanism, which regulates inflammatory and oxidative stress mediators [68]. For example, it functions to trigger inducible nitric oxide synthetase (iNOS) expression, which promotes the production of nitric oxide (NO) by monocytes and macrophages [68].

Juel and colleagues [55, 56] examined the production of NF-*κ*B P50 in unstimulated 10 ng/mL TNF-*α* and 50 ng/mL PMA (phorbol 12-myristate 13-acetate, PMA) stimulated peripheral blood lymphocytes in 18 CFS/ME patients and 18 age-matched controls. Results demonstrated both unstimulated (10 ng/mL) TNF-*α* (*P* = 0.0009) and PMA (0.008) stimulated production of NF-*κ*B to be significantly higher in CFS/ME patients compared to controls. Additionally, positive correlations were reported between the production of NF-*κ*B and severity of illness in CFS/ME patients (as measured by fibro fatigue scale) and with symptoms, including muscular fatigue and tension. The function of p53 is pivotal in the regulation of glycolysis and mitochondrial respiration as it reduces the activity of the glycolytic pathway and stimulates mitochondrial O_2_ consumption and aerobic respiration. However, when inhibited there is a shift to anaerobic glycolysis and reduced O_2_ consumption [65].

Aside from immune dysfunction, several studies have reported mitochondrial dysfunction to be caused by abnormal levels of key mitochondrial enzymes. For example, in a paper by Smits and colleagues [69] a significant reduction in citrate synthase in biopsies (quadriceps region) from patients with CFS/ME was reported when compared to healthy control samples. Citrate synthase is an enzyme located in the mitochondrial matrix, which plays a critical role in the tricarboxylic cycle [70]. Similarly, the McArdle group [35] reported a decrease in citrate synthase as well as succinate reductase and cytochrome- C oxidase (Complex IV), which are two of the four mitochondrial transmembrane enzyme complexes of the electron transport chain. Nevertheless, in contrast to the McArdle group [35], Smits and colleagues [69] attributed the decrease in transmembrane enzymes to be the result of reduced physical activity levels, frequently present in CFS/ME patients as opposed to underlying mitochondrial dysfunction. Nonetheless, a paper by Edwards and colleagues [36] reported there to be no significant difference in partial cytochrome-C oxidase in skeletal muscle biopsies between CFS/ME patients and healthy matched controls.

Evidence also exists to suggest that CFS/ME patients exhibit significantly reduced levels of coenzyme Q10, an important mitochondrial nutrient that functions as a cofactor for the production of ATP in the mitochondria and displays significant antioxidant activity [71]. In a study conducted by Maes and colleagues [37], CFS/ME patients (*n* = 58) displayed significantly lowered plasma coenzyme Q10 concentration compared to healthy controls (*n* = 22). Moreover, in CFS/ME patients there was a significant inverse relationship exhibited between plasma coenzyme Q10 concentration and fatigue severity measured by means of the fibro fatigue scale.

Furthermore, CFS/ME patients may also exhibit alterations in L-carnitine and acylcarnitine homeostasis [72]. L-carnitine is a ubiquitously occurring trimethylated amino acid that plays an important role in the transport of long chain fatty acids across the inner mitochondrial membrane, which is essential for energy production via fatty acid metabolism [72]. Previous studies have reported a reduction in endogenous plasma L-carnitine and total carnitine levels in patients with CFS/ME [73, 74]. Nevertheless, other studies have not always replicated these findings [75, 76]. Reuter and Evans [72] postulated this to be related to the use of varying methodological approaches, with some studies solely focused on free carnitine and total carnitine rather than the level of each individual acylcarnitine, which may be “cancelled out” by normal levels of other acylcarnitines in CFS/ME patients. To overcome this weakness Reuter and Evans [72] utilised tandem mass spectrometry to quantify individual acylcarnitine levels in plasma samples to provide a more detailed carnitine profile. Results demonstrated significant alterations in C8:1, C12DC, C14, C16:1, C18, C18:1, C18:2, and C18:1-Oh acylcarnitines. What is more, significant correlations between acylcarnitine and clinical symptomology were observed.

## 5. Postexertional Malaise and Immune Function

CFS/ME patients report a changeable pattern to their symptoms and physical and cognitive capabilities, often with severe symptom exacerbation following physical exercise [10, 25]. This is termed PEM, with approximately 95% of CFS/ME patients experiencing PEM [77]. As regards the cause of PEM, it has been suggested that exercise may exhibit the ability to amplify preexisting immune abnormalities, in addition to oxidative and nitrosative stress [78]. Immunological abnormalities have been reported following exercise in CFS/ME. For example, observations in CFS/ME symptom flare after moderate intensity exercise have been reported to be directly linked to the levels of interleukin-1*β* (IL-1*β*), IL-12, IL-8, IL-10, and IL-13, 8 hours after exercise [38]. Additionally, sustained increase in plasma TNF-*α* in CFS/ME patients and not in healthy controls has been observed after exercise [38]. Moderate intensity exercise has also been reported to induce a larger 48-hour postexercise area under the curve for IL-10 [39].

However, a recent systematic review [40] compared 23 case control studies regarding exercise-induced immunological changes in CFS/ME patients versus healthy control participants. The authors reported in comparison to healthy participants that CFS/ME patients exhibited a more exaggerated response in the complement system, indicated by C4a split product level and enhanced oxidative stress, combined with a delayed and reduced antioxidant response. Finally, the authors also reported there to be an apparent alteration in immune cell gene expression profile, which was evidenced by an increase in postexercise IL-10 and toll-like receptor 4 gene expression. Nonetheless, in contrast to previous work there was no reported change in circulating pro- and/or anti-inflammatory cytokines. Effectively, the review confirmed CFS/ME patients to respond differently to an exercise-based stimulus, resulting in a more pronounced immune response.

## 6. Muscle Bioenergetic Dysfunction

Even a minimal decrease in muscle pH interferes with cross bridge binding and ATPase activity due to competitive binding and reduced enzyme function [71]. Decreased intracellular pH impairs oxidative enzyme activity and may adversely affect ryanodine receptor function [79]. Recent studies have also confirmed the presence of a peripheral bioenergetic abnormality in CFS/ME patients [15, 41, 63].

In a cross-sectional study conducted by Jones et al. [15] novel MRS techniques were utilised to investigate muscle acid handling following exercise in CFS/ME patients and the relationship with autonomic dysfunction. CFS/ME patients (*n* = 16) and age- and sex-matched normal controls (*n* = 8) performed an exercise protocol, which consisted of 3 minutes of plantar flexion at 35% load maximum voluntary contraction (MVC) at a rate of 0.5 Hz, followed by 3-minute recovery. After the period of exercise, phosphorus magnetic resonance spectroscopy (PMRS) was utilised to investigate intramuscular acid handling. Results demonstrated a significant suppression of proton efflux immediately after exercise (*P* < 0.05) in CFS/ME patients and significantly (*P* < 0.05) prolonged time taken to reach maximum proton efflux. In controls, a strong inverse correlation between maximum proton efflux and nadir pH following exercise (*r* ^2^ = 0.6, *P* < 0.01) was reported. However, in CFS/ME the significance of this relationship was lost (*r* ^2^ = 0.003; *P* = ns). Collectively, these findings demonstrated CFS/ME patients to exhibit abnormalities in the recovery of intramuscular pH following standardised exercise. Effectively, proton efflux is crucial for acidosis resolution, with the immediate postexercise period associated with maximum proton efflux in healthy individuals; however this initial fast phase does not appear to occur in all CFS/ME patients [15]. Furthermore, the authors also acknowledged there to be a close relationship between the degree of intramuscular acidosis and proton efflux, demonstrating a closely regulated process, which has been observed in healthy individuals [80] in addition to the study control group. Nevertheless, they concluded this relationship to be lost in CFS/ME patients. However, the relatively small sample size of this study made it difficult to draw firm conclusions. Therefore, further adequately powered studies are required to investigate the relationship further.

In contrast, Wong et al. [42] reported no difference in intramuscular pH at rest and exhaustion and during early and late recovery, following a graded exercise test to exhaustion. Measurement of intramuscular pH of the gastrocnemius muscle was performed via 31P nuclear magnetic resonance (NMR) spectroscopy. However, the authors did report changes in PCr and pH to occur more rapidly at the onset of exercise in CFS/ME patients compared to controls, which was suggested to be indicative of accelerated glycolysis. Nevertheless, the authors postulated this finding to reflect a lower level of physical endurance due to inactivity in the CFS/ME patient cohort. Nonetheless, it is important to interpret this study with caution, as the CFS/ME patients were able to complete a maximal exercise test to exhaustion. Therefore, it suggests that the patients in this study were not severely incapacitated by the condition and therefore may not be representative of the wider CFS/ME population.

Alternatively, Jones et al. [41] reported prolonged postexercise recovery from acidosis. In this investigation CFS/ME patients and age/sex-matched healthy controls performed a similar exercise protocol (35% MVC plantar flexion for 180 s and 390 s recovery, repeated 3 times). In addition, participants were also required to perform a MVC assessment and a cycle based cardiorespiratory fitness test. Results revealed the ability to divide patients into two distinct groups; 8 (45%) demonstrated normal phosphocreatine (PCr) depletion in response to exercise at 35% MVC, with MVC strength values comparable to controls. In the second grouping, 10 CFS/ME patients exhibited low PCr depletion (generating abnormally low MVC values). Results demonstrated anaerobic threshold (AT), VO_2_, and VO_2_ peak to be significantly reduced in all CFS/ME patients compared to controls. Essentially, one implication of a reduced AT would be a reliance upon anaerobic as opposed to aerobic metabolism, with the predicted consequence of greater short-term acid generation within the muscle as a result of an overutilisation of the lactate dehydrogenase pathway [15]. This was further confirmed by MRS demonstrating CFS/ME patients to exhibit markedly increased intramuscular acidosis compared to controls at a similar work rate following each 3-minute bout, with prolongation (almost 4-fold) in the time taken for pH to recover to baseline, replicating previous findings [15].

Based on the findings the authors suggested that the profound intramuscular acidosis exhibited with repeat exercise to be at least in part related to poor aerobic capacity. This in relation to the physiology of fatigue closely mirrors that observed in patients with the autoimmune disease primary biliary cirrhosis (PBC). PBC exhibits a comparable peripheral pattern and a similar level to fatigue to CFS/ME [81]. In a study by Hollingsworth and colleagues [81] PBC patients exhibited profound and comparable intramuscular acidosis to the CFS/ME patients in the Luin et al. study [53] following the same repeat exercise protocol. However, one pivotal difference between the conditions, which may contribute to the severity of fatigue in CFS/ME, relates to acid homeostasis. In contrast to CFS/ME patients, when PBC patients undergo repeat exercise the extent of acidosis within the muscle decreases with each repeated exercise bout. This may suggest a compensatory mechanism, which operates to resolve excess acidosis. One potential mechanism that may account for this is increased proton flux and the speed of onset of maximum proton excretion with repeat exercise [41, 81]. This mechanism also plays a role in mitochondrial disease whereby increased proton efflux postexercise helps to compensate for decreased aerobic capacity [82]. Nevertheless, it would appear that in comparison to other conditions that exhibit reduced aerobic capacity and acidosis, CFS/ME patients are unable to compensate for an increased reliance upon anaerobic energy pathways during exercise [41].

While the production of protons as a by-product of anaerobic metabolism is a feature of normal metabolism, the body requires mechanisms to effectively manage protons as even small changes in pH dramatically alter enzyme kinetics, decrease muscle function, and cause fatigue [83]. Thus, slow recovery from acidosis in CFS/ME may relate to the ineffective exporting of protons from the recovering muscle. Protons are actively transported out of the muscle by 3 main groups of proton transporters [55, 56]:  Na^+^/H^+^ antiporters, namely, NHE1 [84], sodium/bicarbonate cotransporters (NBCs) [85], and most predominantly monocarboxylate transporters (MCT), whereby in the latter group MCT-1 and MCT-4 isoforms seem to be of particular importance in human skeletal muscle [86]. During rest, intramuscular pH is primarily influenced by NHEs, with MCTs and NBCs playing a greater role during the recovery from muscular contraction. These transporter systems are under autonomic regulation [87]. It is possible that the impaired function of acid transporters occurs in CFS/ME, which may be a consequence of autonomic dysfunction [88–90]. Additionally, it is also possible that reduced vascular runs off (related to autonomic dysfunction), resulting in decreased vascular flow into and out of the muscle following exercise which may have an effect on O_2_ delivery, potentially limiting the function of the three-enzyme complex pyruvate dehydrogenase complex (PDC) [91].

A subgroup of patients (low PCr depletion) exhibited no excess acidosis, which appeared to be entirely the consequence of lower MVC values compared to normal PCr depletion controls. Interestingly, despite markedly lower MVC values the patients perceived themselves to be working maximally immediately following the MVC assessment. However, despite this perception the authors postulated that these findings related to a type of exercise avoidance behaviour. Kinesiophobia is defined as an excessive, irrational, and debilitating fear of movement and activity resulting from a fear of vulnerability to painful injury or reinjury [64, 71] and has been reported to play a role in a variety of musculoskeletal disorders including CFS/ME patients who experience widespread pain [92, 93]. Therefore, the fear of the consequence of an action such as exercise may lead to avoidance behaviour in patients with CFS/ME patients [41]. However, the study was limited, as it did not include a repeat assessment.

Interestingly, previous studies have also reported the existence of subgroups within CFS/ME patient cohorts in relation to glycolytic metabolism and intramuscular pH regulation [18, 20, 42, 43]. However, unlike the studies conducted by Jones et al. [15, 41], the earlier studies have reported an absence in abnormal glycolytic metabolism in the majority of the CFS/ME patients. Barnes et al. [18] explored intramuscular pH regulation in 46 CFS/ME patients via 31P MRS. Results demonstrated no consistent abnormalities in glycolysis or pH regulation at rest or following exercise when the group was taken as a whole. Nonetheless, 12 patients did exhibit abnormal PCr depletion following exercise, with 6 patients within this group displaying increased intramuscular acidification in relation to PCr depletion and the other 6 demonstrating reduced acidification. This study illustrates the heterogeneity within the CFS/ME patient population and suggests that subgroups do exist in CFS/ME that display abnormal glycolytic metabolism and intramuscular pH. Similarly, in Lane et al.'s studies [20, 43] CFS/ME patients completed a subanerobic threshold exercise protocol. Results revealed only a small subgroup (8%) of CFS/ME patients to have an increased blood lactate response to exercise and muscle biopsies revealed a relative increase in type 2 glycolytic fibres for this subgroup.

## 7. Acidosis because of Impaired PDC Function

It is possible that the previously demonstrated muscle cell acidosis in CFS/ME is the consequence of downregulated PDC function and a concomitant increase in the metabolism of pyruvate to lactic acid (overutilisation of lactate dehydrogenase (LDH) pathway). PDC is a 3-protein complex responsible for a series of reactions that convert pyruvate to acetyl coA during aerobic respiration. Principally, when the function of this complex is reduced, pyruvate that has been generated by glycolysis accumulates within the cells and is metabolised anaerobically to lactic acid. This accumulation causes a drop in pH and concurrent deterioration in muscle function [94].

The phenotype of fatigue exhibited by CFS/ME patients closely mirrors that seen in fatigue associated primary biliary cirrhosis patients (PBC). For example, Hollingsworth et al. [81] reported PBC patients to exhibit significant acidosis due to an overutilisation of the lactate dehydrogenase pathway, following a low-level repeat exercise protocol [81]. Furthermore, the authors postulated that the increased dependence on anaerobic pathways of energy production resulted in the fatigue associated with PDC. The idea that impaired PDC function leads to an overutilisation of the lactate dehydrogenase pathway is in agreement with other studies. For example, Murrough et al. [95] reported significantly higher levels of lactate in ventricular cerebrospinal fluid in CFS/ME patients when compared to healthy controls. Similarly, Constantin-Teodosiu and colleagues [96] in an experimental exercise model using rats demonstrated that when PDC function was decreased via the use of PPAR modulating drugs which upregulated PDK function, lactate accumulated intramuscularly which led to decreased muscle function.

Therefore, impaired energy generation in muscle, an increase in the lactate/pyruvate ratio in CFS/ME patients, and a propensity towards excess intramuscular acidosis following limited exercise suggest PDC dysfunction in the muscle of CFS/ME patients, which has implications in relation to the expression of fatigue. Furthermore, as previously discussed CFS/ME patients display significant intramuscular abnormalities relating to both acid generation and clearance from tissue, which has been postulated to relate to a centrally perceived “stop signal,” leading to a disproportionate perception of fatigue [71].

## 8. Abnormal AMPK Activation and Glucose Uptake

A recent study [26] reported striking biochemical differences in skeletal muscle cultures established from 10 CFS/ME patients and 7 age-matched controls. Samples were subjected to electrical pulse stimulation (EPS), for 24 hours, and examined for exercise-associated changes. Key differences emerged, in the basal state; there was increased myogenin expression in CFS/ME samples but a decrease in IL-6 secretion during differentiation when compared to control samples. Following 16 hours of EPS there was a significant increase (*P* < 0.006) in AMP-activated protein kinase (AMPK) phosphorylation and glucose uptake (*P* < 0.0001) in control samples when compared to unstimulated control cultures. Alternatively, CFS/ME samples demonstrated no increase in AMPK phosphorylation or glucose uptake. Nevertheless, glucose uptake remained responsive to insulin, suggesting exercise related dysfunction. Furthermore, IL-6 secretion in response to EPS was significantly reduced (*P* < 0.05 versus corresponding control) across all time points measured.

As regards the function of AMPK, it is a phylogenetically conserved fuel-sensing enzyme, consisting of a heterotrimeric complex composed of a catalytic *α* subunit and regulatory *β* and *γ* subunits [97]. During exercise under normal physiological conditions, AMPK is activated in the skeletal muscle of healthy humans, with exercise suggested to be the most powerful physiological activator of AMPK [98, 99]. Upon activation AMPK sets into motion processes that increase ATP production, such as glucose transport and fatty acid oxidation [97], while decreasing others that consume ATP, for example, lipid and protein synthesis and cell growth and proliferation [97, 99, 100]. Evidence also suggests AMPK to have a broader range of actions including mitochondrial biogenesis [99, 101] and skeletal muscle angiogenesis [102], suggesting AMPK activation to play a key role in peripheral muscle function during exercise.

However, it is important to consider the role of physical activity on AMPK activation. For example, trained subjects have been reported to express higher levels of *α*1 AMPK in comparison to untrained individuals [103]. Furthermore, a 3-week endurance training intervention with young male participants resulted in increases in *α*1 and *α*2 AMPK protein expression in addition to ACC-*β* phosphorylation, which suggested basal activity of AMPK to be increased [104].

Therefore, it should be considered that the decreased AMPK activation reported in CFS/ME muscle samples after EPS might be the result of lowered physical activity levels of the CFS/ME sample donors, when compared to control donors. As regards study recruitment criteria, although participants were age-matched, it was not specified whether any measures had been taken to ensure donors were matched in terms of physical activity habits. Future work is required with patients and controls that are matched in terms of physical activity.

In addition to impaired activation of AMPK, the study also reported reduced IL-6 secretion in response to EPS. Interestingly, previous studies have reported IL-6 to activate AMPK in skeletal muscle by increasing the concentration of cAMP and secondly by increasing the AMP : ATP ratio [105].

The inability of CFS/ME muscle cells to activate AMPK and glucose uptake in addition to reduced IL-6 secretion in response to EPS is suggestive of underlying peripheral muscle dysfunction in CFS/ME. However, further work is required to investigate the mechanisms that lead to impaired activation of AMPK in those with CFS/ME.

## 9. Conclusion

CFS/ME patient perceptions of the nature of their condition frequently cite “peripheral” as opposed to a central origin, with many descriptions of their fatigue regularly referring to difficulty in maintaining muscle activity due to perceived lack of energy, or through muscle fatigue [15, 17]. There is increasing evidence to suggest that muscular biochemical abnormality may play a major role in CFS/ME associated fatigue. The literature suggests patients to exhibit profound intramuscular dysfunction regarding acid generation and clearance, with a tendency towards an overutilisation of the lactate dehydrogenase pathway following relatively low-level activity. However, the precise mechanisms underlying the dysfunction are yet to be fully elucidated. There is a real need for adequately powered studies to examine PDC function in vitro, to determine the mechanisms responsible for bioenergetic dysfunction and peripheral fatigue.

## Figures and Tables

**Table 1:** Summary of various proposed mechanisms underlying muscle dysfunction in CFS/ME.

Proposed mechanism underlying muscle dysfunction in CFS/ME	Study
*Central sensitisation*	
Decreased pain threshold in patients.	Whiteside et al. (2004) [25], Meeus et al. (2010) [27]
Generalised hyperalgesia.	Nijs et al. (2012) [28], Vecchiet et al. (2003) [29]

*Oxidative and nitrosative stress*	
Elevated TBARS prior to exercise in patients with history of severe infection.	Jammes et al. (2013) [22]
Elevated TBARS in patients with history of stress factors.	Jammes et al. (2012) [30]
Oxidative damage to endogenous epitopes, autoimmunity, and links with muscle fatigue.	Maes et al. (2006) [31]

*Mitochondrial dysfunction*	
Mitochondrial function influenced by increased immune-inflammatory stress pathways in patients.	Broderick et al. (2010) [32]
Reduction in Th1 and Th17 function and movement toward Th2 dominant immunity.	Brenu et al. (2011) [33]
Reduction in mitochondrial enzyme level citrate synthase in patient muscle samples.	Skowera et al. (2004) [34]
Reduction in mitochondrial enzymes succinate reductase and cytochrome-C oxidase in skeletal muscle of patients.	McArdle et al. (1996) [35]
No difference in cytochrome-C oxidase levels in muscle biopsies.	Edwards et al. (1993) [36]
Patients reported to exhibit reduced levels of coenzyme Q10, with a significant inverse relationship between plasma coenzyme Q10 and fatigue severity measured via fibro fatigue scale.	Maes et al. (2009) [37]

*Postexertional malaise (PEM) and immune function*	
Moderate intensity exercise and symptom flare (PEM) in patients, directly linked to IL-*β*, IL-12, IL-8, IL-10, and IL-13. Increased TNF-*α* postexercise in patient cohort postexercise.	White et al. (2004) [38]
Moderate intensity exercise also reported to induce a larger 48-hour postexercise area under the curve for IL-10 in patients.	Light et al. (2012) [39]
In comparison of 23 case control studies no evidence of a significant change in circulating pro/anti-inflammatory cytokines was reported. However, exaggerated complement system response, indicated by C4C split product level, enhanced oxidative stress and combined delayed and reduced antioxidant response.	Nijs et al. (2014) [40]

*Muscle bioenergetic dysfunction*	
Evidence of significant (*P* < 0.05) suppression in proton efflux immediately after exercise and significantly prolonged time to reach maximum proton efflux following low-level exercise (plantar flexion, 35% MVC).	Jones et al. (2010) [15]
Prolonged postexercise recovery from exercise in patients, indicated by marked increase in intramuscular acidosis compared to controls at a similar work rate. After each 3-minute bout of exercise (plantar flexion, 35% MVC), a 4-fold increase in the time taken to recover to baseline.	Jones et al. (2012) [2, 41]
No difference in intramuscular pH at rest, exhaustion, and early or late recovery following graded exercise to exhaustion. However, evidence of accelerated glycolysis at onset of exercise was illustrated by more rapid PCr depletion.	Wong et al. (1992) [42]
No consistent abnormalities in pH regulation following exercise when patient cohort is taken as a whole. However, 6 patients exhibited increased intramuscular acidification in relation to PCr depletion.	Barnes et al. (1993) [18]
In subanaerobic threshold exercise protocol only small subgroup of patients reported to have increased blood lactate responses to exercise.	Lane et al. (1998) [20, 43]

*Abnormal AMPK activation and glucose uptake*	
No increase in AMPK phosphorylation or glucose uptake 16 hours following electrical pulse stimulation in CFS/ME patients. Compared to significant increases in both parameters in control participants.	Brown et al. (2015) [26]
